# Colorectal Cancer: Epidemiology, Risk Factors, and Prevention

**DOI:** 10.3390/cancers16081530

**Published:** 2024-04-17

**Authors:** Gholamreza Roshandel, Fatemeh Ghasemi-Kebria, Reza Malekzadeh

**Affiliations:** 1 Golestan Research Center of Gastroenterology and Hepatology, Golestan University of Medical Sciences, Gorgan 49178-67439, Iran; roshandel@goums.ac.ir (G.R.); kebria_fgh@goums.ac.ir (F.G.-K.); 2 Digestive Oncology Research Center, Digestive Diseases Research Institute, Tehran University of Medical Sciences, Tehran 14117-13135, Iran

**Keywords:** colorectal cancer, epidemiology, risk factors, prevention, screening

## Abstract

**Simple Summary:**

In 2020, more than 1.9 million cases of colorectal cancer (CRC) occurred, and almost 0.9 million patients died due to CRC throughout the world. There are differences in distribution and time variations in CRC between different countries. This diversity is mainly due to differences in risk factors among populations. CRC may be prevented by primary and secondary prevention methods. Primary prevention includes avoiding risk factors, e.g., alcohol consumption, and increasing protective factors, e.g., physical activity. The secondary prevention method, called CRC screening, consists of diagnosis and treatment of precancerous lesions of the colorectum. Although a large amount of evidence is available for different aspects of CRC, controversies remain regarding the most important factors and most effective control programs for CRC in different populations. In this review, we will present the most updated evidence regarding CRC’s distribution, related factors, and preventive methods.

**Abstract:**

Colorectal cancer (CRC) is the third most common cancer and the second most common cause of cancer mortality worldwide. There are disparities in the epidemiology of CRC across different populations, most probably due to differences in exposure to lifestyle and environmental factors related to CRC. Prevention is the most effective method for controlling CRC. Primary prevention includes determining and avoiding modifiable risk factors (e.g., alcohol consumption, smoking, and dietary factors) as well as increasing protective factors (e.g., physical activity, aspirin). Further studies, especially randomized, controlled trials, are needed to clarify the association between CRC incidence and exposure to different risk factors or protective factors. Detection and removal of precancerous colorectal lesions is also an effective strategy for controlling CRC. Multiple factors, both at the individual and community levels (e.g., patient preferences, availability of screening modalities, costs, benefits, and adverse events), should be taken into account in designing and implementing CRC screening programs. Health policymakers should consider the best decision in identifying the starting age and selection of the most effective screening strategies for the target population. This review aims to present updated evidence on the epidemiology, risk factors, and prevention of CRC.

## 1. Introduction

With more than 1.9 million new cases and 0.9 million deaths in 2020, colorectal cancer (CRC) was the third most common cancer and the second most common cause of cancer mortality worldwide [1,2,3]. Geographical disparities were reported in incidence and mortality rates, time trends, and the future burden of CRC across different countries and regions [1,2,3]. There were also changes in the age pattern of CRC, with increasing incidence in the young, especially in developed countries [4]. These disparities may reflect differences in exposure to the risk factors for CRC, including lifestyle and environmental factors [5,6]. Identifying and avoiding modifiable risk factors, especially lifestyle factors (e.g., alcohol consumption, smoking, obesity, unhealthy diet) as well increasing protective factors (e.g., physical activity, taking specific medications such as aspirin, healthy diet) [5,6] have a pivotal role in the primary prevention of CRC. CRC screening (secondary prevention) for the detection and removal of premalignant lesions of the colorectum is also considered an effective preventive method in CRC control programs [7,8,9]. Despite large numbers of studies being conducted on different aspects of CRC, there are still challenges regarding the most important risk factors and most effective preventive strategies for CRC in different populations. An updated, concise review of all of this information may be helpful for researchers, physicians, and policymakers. In this review, we performed a comprehensive search of the latest available literature to present the most updated evidence on the epidemiology, risk factors, and prevention of CRC.

## 2. Epidemiology

### 2.1. Mortality

According to the GLOBOCAN estimates provided by the International Agency for Research on Cancer (IARC), colorectal cancer (CRC) was the second most common cause of cancer-related death (after lung cancer) worldwide, with an estimated number of 935,173 deaths and an age-standardized mortality rate (ASMR) of 9.0 per 100,000 person-years in 2020. The highest ASMR of CRC was estimated for Europe (12.3), and the lowest one was reported for Africa (5.6) and the eastern Mediterranean region (EMRO) (5.3). The rates were almost comparable for other regions, including Oceana (9.3), Asia (8.6), Latin America (8.2), and North America (8.2) [2,10]. The GLOBOCAN estimates suggested a higher ASMR for CRC in men (11.0) than women (7.2), with 515,637 and 419,536 deaths in men and women, respectively [2,10].

### 2.2. Incidence

**Figure 1:**
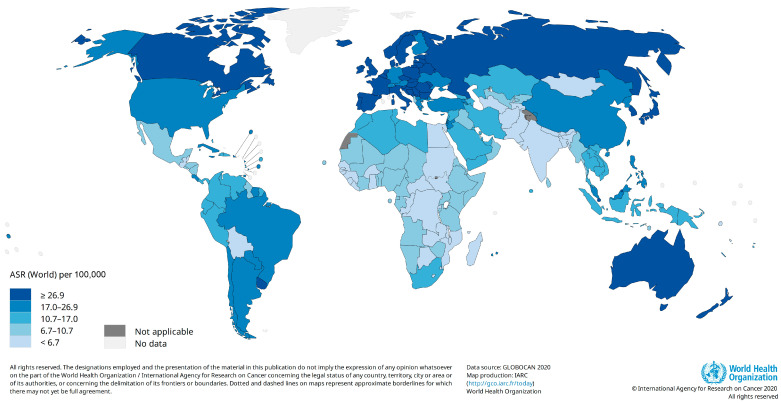
Estimated age-standardized incidence rate for colorectal cancer, 2020 (Data source: GLOBOCAN 2020; Map production: IARC, http://gco.iarc.fr/today, accessed on 10 October 2023).

CRC was reported as the third most common cancer worldwide (after breast and lung cancers) in 2020. The number of new cases of CRC was estimated to be 1,931,590 with an age-standardized incidence rate (ASR) of 19.5 per 100,000 person-years. The highest ASRs were estimated for Europe (30.4), followed by Oceana (29.8) and North America (26.2). After Asia (17.6) and Latin America (16.6), EMRO (9.1) and Africa (8.4) had the lowest ASRs for CRC (Figure 1). Hungary (45.3) and Guinea (3.3) had the highest and lowest ASRs for CRC among the other countries. The ASRs for CRC were almost four-fold higher in high-income (30.2) countries compared with low-income (8.8) and low–middle-income (7.4) countries. Similar discrepancies were reported in the incidence rates for CRC between countries with very high human development indices (HDIs) (29.4) and high HDIs (20.4) and those with medium HDIs (6.1) and low HDIs (7.4) [2,10]. This diversity may be explained by differences in exposure to CRC risk factors. We will discuss CRC risk factors in the next sections of this paper.

### 2.3. Time Trends and Future Burden

**Figure 2:**
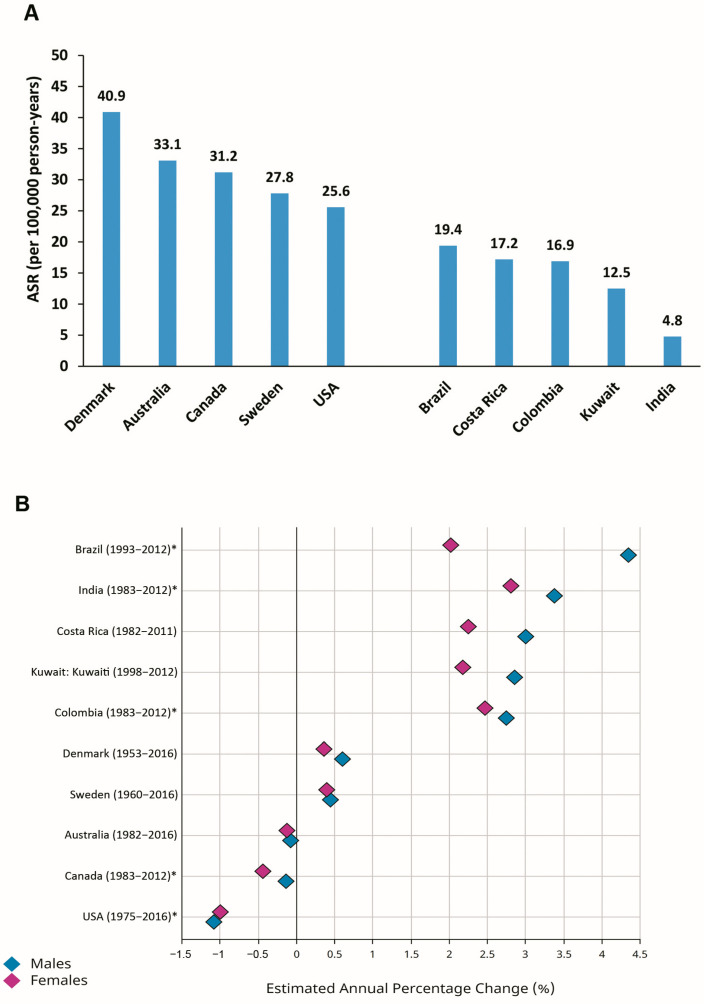
Age-standardized incidence rates (ASRs) (per 100,000 person-years) in 2020 (**A**) and estimated annual percentage changes (EAPCs) in ASRs (**B**) for colorectal cancer in selected transitioned (Denmark, Australia, Canada, Sweden, USA) and transitioning (Brazil, Costa Rica, Colombia, Kuwait, India) countries (Source: GLOBOCAN 2020; Figure production, B: IARC, http://gco.iarc.fr/overtime, accessed on 23 March 2023) (* subnational data).

There are discrepancies in the pattern of time trends in the incidence of CRC worldwide [1,6,11,12,13]. Considering the estimated annual percentage changes (EAPCs) in the ASRs for CRC during recent decades, constant or even slightly declining trends have been reported for formerly high-risk (transitioned) countries, including Denmark, Sweden, Australia, Canada, and the USA (Figure 2). In contrast, transitioning countries with relatively low-risk of CRC, including Brazil, Costa Rica, Colombia, Kuwait, and India, have shown increasing trends in the ASRs for CRC during the last decades (Figure 2) [1,11,14].

According to the GLOBOCAN 2020 estimates, the number of new cases of CRC will increase from 1,931,590 in 2020 to 3,154,674 in 2040, worldwide, suggesting a 63.3% increase in the number of new CRC cases. The greatest changes were estimated for currently low-risk population regions, including Africa (95.0%), EMRO (92.0%), Latin America (74.0%), and Asia (70.8%). The estimated changes in the number of new CRC cases are considerably lower in very-high-HDI countries (35.0%) compared to countries with low (10.2.6%), medium (72.1%), and high HDIs (67.9%) [3]. The increasing trends in the incidence rates for CRC in low-risk populations may be mainly explained by the increasing prevalence of risk factors, which will be discussed in detail in the next sections of this paper [6,15,16,17].

### 2.4. Early-Onset CRC

**Figure 3:**
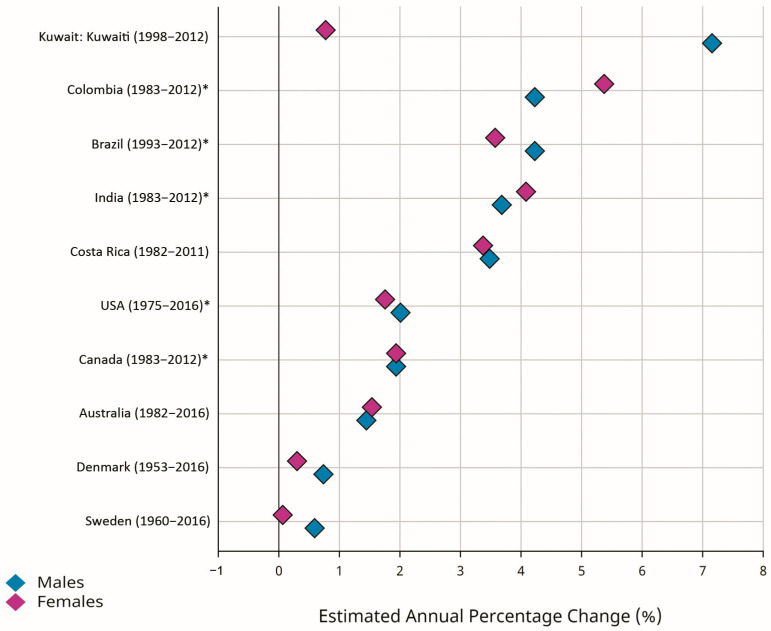
Estimated annual percentage changes (EAPCs) of the age-standardized incidence rates (ASRs) for early-onset colorectal cancer (EOCRC) (age 0–49 years) in selected developed (Denmark, Australia, Canada, Sweden, USA) and developing (Brazil, Costa Rica, Colombia, Kuwait, India) countries (Source: GLOBOCAN 2020; Figure production: IARC, http://gco.iarc.fr/overtime, accessed on 23 March 2023) (* subnational data).

Early-onset CRC (EOCRC) occurs in individuals younger than 50 years old. According to the GLOBOCAN estimates, there were 188,069 new cases of EOCRC, with an ASR of 2.9 per 100,000 person-years worldwide. The ASRs for EOCRC were 3.0 and 2.7 per 100,000 person-years in men and women, respectively. The highest ASRs for EOCRC were estimated for North America (6.1) and Oceania (5.3), while Africa (2.0), Asia (2.6), and Latin America (2.9) were reported as low-risk areas [2,10]. Recent evidence suggested an increasing incidence rate for EOCRC in different populations, with greater changes in developing countries [1,5,18,19,20,21,22,23,24,25]. Figure 3 shows greater EAPCs for EOCRC in selected developed countries (Denmark, Australia, Canada, Sweden, USA) and developing countries (Brazil, Costa Rica, Colombia, Kuwait, India). Despite the declining trend in the incidence of CRC in the total population of the developed countries, the incidence rates for EOCRC were increasing both in the developed and developing world. The reasons for increasing trends in the incidence of EOCRC may include genetic predispositions [4,26,27,28,29,30] and exposure to environmental and lifestyle-related factors, including hyperlipidemia, obesity, alcohol consumption, metabolic syndrome, ulcerative colitis, low physical activity, low vitamin D intake, high red meat intake and high sugar-sweetened-beverage intake [28,31,32,33,34,35,36,37]. Recent evidence also suggests that changes in the types and diversity of the species of intestinal microbiota may be associated with increasing trends in the incidence of EOCRC [38,39]. Regarding the increasing trends in the incidence rates for EOCRC and the predicted future burden, it should be considered as a top priority, and further investigations are warranted to clarify its risk factors and to develop and implement the most effective control strategies in all populations worldwide.

## 3. Risk Factors

CRC risk factors, including environmental and genetic factors, may be divided into modifiable and non-modifiable risk factors [40,41,42,43,44].

### 3.1. Modifiable Risk Factors

Modifiable risk factors for CRC may be controlled by effective risk-factor reduction measures and therefore are of special interest to policymakers for designing CRC control programs.

#### 3.1.1. Alcohol Consumption

Various reports from different populations suggest a positive association between alcohol consumption and the risk of CRC [45,46,47,48,49,50]. Cai et al. suggested a proportional positive association between alcohol consumption and the risk of CRC, with the greatest risk in heavy drinkers (≥50 g/day of ethanol) [47]. In a pooled cohort study and Mendelian randomization analysis, Zhou et al. reported that drinking alcohol was causally associated with an increased CRC risk with an odds ratio of 1.79 (95% CI: 1.23–2.61). The findings of this study also revealed that the pathogenic effect of alcohol on CRC could be partly attributed to DNA methylation by regulating the expression of specific genes [49].

#### 3.1.2. Smoking

The direct association between smoking and the risk of CRC was consistently reported by a large number of studies on different populations, suggesting significant dose–response effects and a reduction in CRC risk after smoking cessation [51,52,53,54,55]. By inhaling toxic chemicals in smokers, the colorectal mucosal cells are exposed to well-known carcinogens, including nitrosamines, heterocyclic amines, polycyclic aromatic hydrocarbons (PAH), and benzene. Long-term exposure to these carcinogens will result in genetic and molecular changes in colorectal cells, and, finally, the accumulation of these pro-oncogenic changes may cause the development of CRC [56,57].

#### 3.1.3. Obesity

Reports from different studies, including large-scale cohort studies, suggest a direct association between obesity and CRC, with associations in men and women [58,59,60,61,62,63,64,65]. The association between obesity and the development of CRC may be explained by different mechanisms, mainly by the effects of pro-inflammatory cytokines (e.g., interleukin-6 and tumor necrosis factor alpha) and insulin or insulin-like growth factor on proliferation of tumor cells in obese individuals [61,63,64]. High levels of bile acids in obese individuals may increase inflammatory processes by the destruction of colorectal epithelial cells, resulting in progression to CRC [66,67].

#### 3.1.4. Sedentary Lifestyle

The association between the risk of CRC with low physical activity or sedentary lifestyle was reported in different populations. The results of a study on data from the Asia-Pacific Cohort Studies Collaboration suggested that any physical activity was associated with 0.25 to 0.30 reduction in the hazard of CRC mortality [68]. The inverse association between physical activity and CRC incidence was suggested by several observational studies with RR reduction of 0.30 to 0.50 and similar findings in men and women [69,70,71]. The association between sedentary lifestyle and CRC risk may be partly explained by higher rates of obesity, higher plasma glucose levels, insulin resistance, and abnormal intestinal peristalsis in individuals with sedentary lifestyle [72]. Further investigations, especially clinical trials, are needed to clarify this association.

#### 3.1.5. Unhealthy Diet (High Intake of Red and Processed Meat and Fat)

The results of a recent umbrella review of 45 meta-analyses suggested an association between dietary habits and the risk of CRC [73]. High intake of red and processed meat was consistently reported as a risk factor for CRC incidence [74,75,76]. Each 100 g/day increase in dietary red meat and each 50 g/day increase in dietary processed meats may increase the risk of CRC by 0.10–0.16 and 0.16–0.22, respectively [77,78]. Cooking meat, especially at high temperatures (e.g., grilling or barbecuing), may result in the production of various carcinogens, including heterocyclic aromatic amines and polycyclic aromatic hydrocarbons. In addition, the processing of meat (e.g., curing or smoking) may cause the formation of different carcinogens, including N-nitroso compounds and polycyclic aromatic hydrocarbons [74]. High intake of red and processed meat will expose the colorectal mucosa to these carcinogens, resulting in an increase in the risk of CRC [72]. High intake of dietary fat, especially from animal sources, was also suggested as another dietary risk factor for CRC [79,80], although another meta-analysis reported no association between dietary fat intake and the risk of CRC [81].

#### 3.1.6. Psychological Stress

There is evidence of the direct association between different types of stress (e.g., work stress) and the risk of CRC [82,83,84,85,86]. The results of a recent meta-analysis suggested a 0.36 increase in CRC risk in those with high levels of work stress [83]. These findings were not supported by the results of other studies [87,88]. These inconsistent findings may be explained by differences in definitions and methods for the measurement of the levels of stress in different studies. Stress may cause overactivation of the hypothalamic–pituitary–adrenal axis, which in turn will result in psychological consequences, including immune system impairment, abnormal metabolic activities, and cancer [89,90]. The results of animal studies suggest associations between increased levels of norepinephrine, epinephrine, and cortisol (due to stress) and cancer initiation [91,92]. Chronic psychological stress may affect different phases of the process of tumorigenesis, including genome instability and mutation, tumor promoting, resistance to cell death, sustained proliferative signaling, induction of angiogenesis, and activation of invasion and metastasis [93]. Due to the increasing prevalence of psychological stress, as well as increasing trends in the incidence of CRC in different populations, further investigations are needed to clarify the association between stress and CRC risk.

### 3.2. Non-Modifiable Risk Factors

Although non-modifiable risk factors cannot be controlled, policymakers are interested in these risk factors as well. Non-modifiable risk factors may be considered for identifying high-risk individuals or populations as candidates for taking preventive interventions.

#### 3.2.1. Age

Advancing age is a major risk factor for CRC incidence. Individuals older than 50 years are specifically at high risk, consisting of more than 90% of all CRC cases [2,10].

#### 3.2.2. Gender

The risk of CRC is higher in men (ASR = 23.4 per 100,000) than women (16.2 per 100,000), with a male-to-female ratio of 1.4 [2,10]. The male-to-female ratio for CRC incidence is greater in high-income (1.4) than low-income countries (1.2) [2,10].

#### 3.2.3. Genetic Predisposition

The most common forms of genetic-related (hereditary) CRC include germline mutations in the APC gene (familial adenomatous polyposis) [94,95] and germline mutations in DNA mismatch repair genes (Lynch syndrome) [96,97,98,99]. Although individuals with these germline mutations are at a much higher risk of CRC [100], they account for only five percent of CRC cases [96]. A higher proportion (16%) of inherited syndromes was reported in EOCRC cases [101,102]. Recent genome-wide association studies (GWAS) identified the association between new genetic variants and the risk of CRC, suggesting the need for further investigation to clarify their clinical implications [103,104]. According to these findings, future resequencing studies may identify rarer variants (e.g., prevalence 0.05–5%) [103].

#### 3.2.4. Family History of CRC

A large number of studies with different designs and methodologies for various populations reported higher risk of CRC in first-degree relatives of CRC patients, with RRs of 2 to 4 [94,96]. A history of CRC [94,96,105,106] or adenomatous colonic polyps [107,108] in first-degree relatives, as important risk factors for CRC, is considered for determining high-risk groups in CRC control programs and guidelines.

#### 3.2.5. Abdominopelvic Radiation

Receiving abdominopelvic radiation therapy in cancer survivors (e.g., those with a history of prostate cancer) was suggested as a risk factor for developing gastrointestinal cancers, especially CRC [109,110,111,112,113,114]. Some guidelines suggest CRC screening at earlier ages or at a specific intervals after cessation of radiotherapy [115].

#### 3.2.6. Personal History of Other Diseases

Specific comorbidities were suggested as risk factors for developing CRC. Patients with inflammatory bowel disease (IBD), including ulcerative colitis or Crohn disease, are at high risk of CRC [116,117,118,119]. Higher risks of CRC were also reported in patients with other comorbidities, including cystic fibrosis [120,121], renal transplantation [122], cholecystectomy [123], coronary heart disease [124], bacterial and viral infections (e.g., human papilloma virus, Helicobacter pylori) [125,126,127,128], antibiotic use [129,130], and diabetes mellitus and insulin resistance [131,132,133]. The association between type 2 diabetes and CRC may be mainly explained by the stimulating effects of insulin (as an important growth factor) on colonic tumor cells, suggesting that pharmacological or lifestyle interventions that lower circulating insulin levels may be beneficial in preventing CRC [134].

#### 3.2.7. Intestinal Microbiota

The gut microbiome, called the “forgotten organ” and composed of a large population of microorganisms, has a complex association with the development of CRC. Increasing evidence suggests changes in the gut microbiome and a reduction in its diversity are closely related to CRC incidence [135,136,137]. Dai et al. and Wong et al. reported the predominance of specific species in the intestinal microbiomes of CRC patients [137,138]. Recent studies provide evidence on CRC-related species of the gut microbiota [139,140]. Ma et al. performed a Mendelian randomization analysis to assess the relationship between gut microbiota and CRC [141]. They found a negative correlation between the *Lachnospiraceae* species and CRC risk, while the *Porphyromonadaceae* species, *Lachnospiraceae* UCG010 genus, Lachnospira genus, and Sellimonas genus had a positive relationship with the risk of CRC. Their findings suggested causal relationships between the intestinal microbiome and the risk of CRC. The results of this study revealed that dysbiosis of the intestinal microbiota may play an important role in development of CRC, suggesting their potential implications in CRC prevention [141]. Microbiome modulation was proposed as a new strategy for the prevention or treatment of CRC [136]. The relationship between gut microbiota and CRC risk may be partly explained by the role of the microbiome in metabolic activities (including the generation and regulation of bile acids, metabolism of amino acids, and carbohydrate fermentation) and the carcinogenic effects of their metabolic derivatives [142]. Further studies are warranted to clarify different aspects of the relationships between intestinal microbiota and CRC risk and their implications in CRC control programs.

### 3.3. High-Risk Groups for CRC

Considering the available evidence on CRC risk factors, high-risk groups for CRC include individuals with a personal history of adenomatous polyps or CRC, a family history of CRC, those with hereditary CRC syndrome (e.g., FAP, HNPCC), those with a personal history of IBD, and a history of abdominal or pelvic radiation [143,144]. Identifying the high-risk individuals/groups for CRC will help researchers and health policymakers to design targeted preventive methods and develop effective CRC control programs.

## 4. Prevention

Preventive methods for CRC may be classified into two main categories, namely primary prevention methods and secondary prevention methods. The primary prevention methods include avoiding CRC risk factors and increasing protective factors for CRC. The secondary prevention methods, called CRC screening, consist of methods for the diagnosis and removal of the precancerous lesions of CRC, called neoplastic colorectal polyps (e.g., colorectal adenomas).

### 4.1. Primary Prevention

#### 4.1.1. Avoiding Risk Factors

Reduction in or elimination of exposure to modifiable risk factors for CRC (Section 2.1) may be associated with a reduction in CRC risk [70]. Cessation of smoking for more than 20 years was reported to be associated with a more than 50% decrease in the odds for different types of colorectal adenomas [145]. Interventions for the restriction of fat intake could reduce the risk of CRC in healthy individuals [146]. Further investigations, especially clinical trials, are warranted to clarify the effects of risk factor reduction on CRC incidence.

#### 4.1.2. Increasing Protective Factors

There is consistent evidence suggesting that protective factors (e.g., dietary factors, physical activities, specific drugs/supplements) are associated with a decreased risk of CRC. We will review recent evidence on the association between the most important protective factors with CRC risk reduction.

Physical activity: There is substantial and consistent evidence on the protective effects of physical activity in CRC [71,147]. Different types of regular physical activity were associated with a 25–30 percent reduction in the risk of CRC [147,148,149,150,151,152]. Performing higher amounts (any amount is better than none) of aerobic physical activity with moderate to vigorous intensity during leisure time was suggested as the optimal characteristic for physical activity for the prevention of CRC [151].

Dietary factors: Different studies suggested protective effects of diets high in fruits and vegetables in CRC [153,154,155,156,157], with about a 50% decrease in the risk of CRC in individuals with the highest intake [158,159]. The risk of CRC was significantly lower in individuals with vegetarian diets compared with non-vegetarians [72,160,161]. Dairy products are another dietary factor for which CRC protective effects were consistently reported [72,73,162]. About a 10% reduction in CRC risk was reported in individuals with daily intakes of 400 g of dairy products or a daily milk intake of 200 g/day [163,164].

Dietary fiber was suggested as another protective factor from CRC. Higher intake of fiber was associated with decreased risk of CRC [72,73,165], with a 10 percent decrease in CRC risk reported for every 10 g/day increase in dietary fiber intake [15,166].

Possible protective effects were also reported (in observational studies) for other dietary factors, including fish [167], garlic [15], and coffee [168,169]. However, the findings were not consistent [157,170,171,172,173,174] and need to be confirmed in prospective interventional studies.

Chemoprevention: The preventive effects of different drugs or supplements in CRC, called chemoprevention, were documented in several studies, especially on average- and high-risk populations [175]. There is substantial consistent evidence on the protective effects of aspirin and other nonsteroidal anti-inflammatory drugs (NSAIDs) in colorectal adenoma and CRC [176,177,178,179]. Several studies reported protective effects of postmenopausal hormone therapy in CRC, with more consistent findings for combined drugs (estrogen plus progestin) [180,181,182]. The protective effects of oral contraceptives were also reported in premenopausal women [183]. Findings from a systematic review of 126 articles suggested protective effects for statins in CRC [184]. Regarding the adverse events of aspirin (e.g., bleeding), especially in older individuals [185], further investigations are warranted to clarify the preventive effects of statins in CRC. Increased intake of calcium and vitamin D [186,187,188] were also proposed as protective factors for CRC, although these findings were not consistent with the results of other studies [189,190,191], suggesting the need for further solid evidence.

### 4.2. Secondary Prevention (CRC Screening)

The process of secondary prevention of CRC (CRC screening) consists of the diagnosis and treatment (removal) of premalignant (adenomatous) polyps of the colon and rectum. There is consistent evidence for the effects of polyp removal on reducing the risk of CRC [192,193]. The results of a recent meta-analysis suggested that different screening methods could significantly reduce CRC-specific mortality [193]. The Fecal Occult Blood Test (FOBT) and lower-GI endoscopy (sigmoidoscopy/colonoscopy) are the main methods for CRC screening. Based on the method for detecting occult blood in stool, the FOBT may be categorized into two groups, including the guaiac-based FOBT (guaiac test) and the fecal immunohistochemical (FIT)-based FOBT (FIT test).

#### 4.2.1. Guaiac-Based FOBT (Guaiac Test)

The guaiac-based FOBT may detect both human and non-human (e.g., from dietary sources) hemoglobin. This is the major limitation of this test, resulting in a high proportion of false positives and low positive predictive values [194,195]. The high-sensitivity guaiac-based FOBT was introduced to improve the sensitivity of the guaiac-based FOBT, although the limitation of low specificity remains, suggesting that policymakers should replace this method with more accurate methods in population-based screening programs [196,197,198,199].

#### 4.2.2. FIT-Based FOBT (FIT Test)

The FIT test identifies only intact human hemoglobin and does not detect non-human hemoglobin (e.g., from dietary sources) and also does not detect digested human hemoglobin (e.g., bleeding from upper gastrointestinal tract or respiratory tract). This advantage of the FIT test (over the guaiac test) resulted in better indices of accuracy for the FIT test in detecting colorectal neoplasia [196,197,200,201,202]. The results of a retrospective cohort study suggested high adherence to a programmatic (e.g., annual) FIT test, resulting in detection in the majority (more than 80%) of patients with CRC diagnosed within one year of testing [203]. Therefore, the FIT test is a non-invasive CRC screening method with acceptable accuracy and high adherence to programmatic testing.

#### 4.2.3. Stool DNA Test

Stool DNA tests detect CRC-associated gene mutations in cells shed from the colorectal epithelium into stool [204,205]. Stool DNA tests were proposed as non-invasive accurate methods for detecting colorectal neoplasia [206,207,208,209], especially when combined with the FIT test (sDNA-FIT test) [210].

#### 4.2.4. Sigmoidoscopy

Examination of the distal colon with the flexible fiber-optic sigmoidoscope (sigmoidoscopy) was suggested as an accurate and acceptable method for detecting premalignant colorectal neoplasia. Different clinical trials on sigmoidoscopy screening reported 20 to 30 percent decreases in CRC incidence and mortality, with sustainable effects over the long term, suggesting sigmoidoscopy as an effective method for CRC screening [211,212,213,214,215,216].

#### 4.2.5. Colonoscopy

The results of different studies suggest an association between colonoscopy examination and a reduction in CRC incidence and mortality [192,216,217,218]. A recent randomized, controlled trial also reported significant effects of colonoscopy examination in reducing CRC risk (RR = 0.69) and mortality (RR = 0.50) [219]. Colonoscopy may be considered as the only CRC screening modality for specific (high-risk) populations or may be used in combination with other non-invasive modalities in general (average-risk) populations [220,221,222].

#### 4.2.6. Computed Tomographic Colonography (CTC)

CTC includes the examination of the abdominal CT scan images of the colon and rectum, simulating the effect of a colonoscopy. The results of different studies suggest high accuracy of CTC for detecting colorectal neoplasia, especially large adenomas [223,224,225,226]. Different factors may affect the accuracy, and especially the sensitivity, of CTC, including the expertise of the radiologist, the mode of imaging (2-D or 3-D), and the characteristics of the scanner and detector. These factors should be mentioned when considering CTC for CRC screening.

### 4.3. Combination and Risk-Based Strategies

A combination of the FOBT and lower-GI endoscopy (sigmoidoscopy/colonoscopy) could increase the accuracy of screening programs for the detection of colorectal neoplasia, suggesting combination methods as the most effective CRC screening method [227,228]. In addition, considering risk stratification to identify high-risk individuals was suggested to increase the effectiveness of CRC screening programs. Different risk factors, including age, gender, smoking, and family history, may be considered for the calculation of risk scores [229,230,231,232]. Combining the risk score with the FIT test was suggested as an effective strategy for more accurate detection of colorectal neoplasia in asymptomatic individuals [233,234,235].

### 4.4. CRC Screening Guidelines

Different guidelines have been developed for CRC screening in high- and average-risk individuals. The screening methods for high-risk groups are specified according to the underlying conditions (e.g., presence of hereditary syndromes such as FAP or HNPCC, family history of CRC, etc.), but most guidelines suggest colonoscopy as the first choice for CRC screening in high-risk groups [236,237,238].

Guidelines on CRC screening in average-risk populations have been issued by different organizations [7,8,9,143,239,240]. In its 2018 guideline, the American Cancer Society (ACS) suggested starting CRC screening in individuals between 45 and 75 years old. Based on the ACS 2018 guideline, CRC screening programs may include annual FIT tests, guaiac-based FOBTs annually, stool DNA tests every 3 years, sigmoidoscopy every 5 years, CTC every 5 years, and colonoscopy every 10 years [143]. The recent (2021) U.S. Preventive Services Task Force (USPSTF) guideline also recommended CRC screening in average-risk populations aged 45–75. According to the USPSTF 2021 guideline, the following modalities are recommended for CRC screening: the guaiac-based FOBT or FIT test every year, sDNA-FIT test every 1 or 3 years, sigmoidoscopy every 5 years, CTC every 5 years, sigmoidoscopy every 10 years plus FIT test every year, and colonoscopy every 10 years [144,239]. The American College of Physicians (ACP) issued its updated CRC screening guideline in 2019. The ACP recommends CRC screening in average-risk adults between 50 and 75 years old. The screening modalities and intervals recommended by the ACP include the FIT test or guaiac-based FOBT every 2 years, sigmoidoscopy every 10 years plus FIT test every 2 years, or colonoscopy every 10 years [8,240].

All organizations emphasize that different factors both at the individual and community levels (e.g., patient preferences, availability of screening modalities, costs, benefits, and adverse events) should be taken into account in the designing and implementation of CRC screening programs. Policy makers are encouraged to pay especial attention to making the best decision in identifying the starting age and selection of the most effective screening modalities for the target population [7,8,9,143,144,239,240,241].

## 5. Conclusions

CRC is the third most common incident cancer and the second most common cause of cancer mortality worldwide. Individuals residing in developed countries are at higher risk of CRC, while the residents of the developing world are at lower risk for this cancer. There were declining trends in the ASR of CRC in developed countries during the last decades, while the trends in developing countries were increasing. In addition, the results of the future predictions (by 2040) show that the greatest increases in the incidence of CRC are predicted to occur in currently low-risk populations, including those in developing countries. Despite the declining trend in the incidence of CRC in the total population of the developed countries, the incidence rates of EOCRC (age below 50 years) were increasing both in the developed and developing world, suggesting the need for further investigations on its risk factors and control methods worldwide.

The most important modifiable risk factors for CRC include alcohol consumption, smoking, obesity, sedentary lifestyle, high intake of red and processed meat and fat, and psychological stress. Age, gender, genetic predisposition, family history of CRC, abdominopelvic radiation, personal history of IBD, and intestinal microbiota were proposed as non-modifiable risk factors for CRC. Policymakers may consider these risk factors for designing risk-based CRC preventive strategies. Further investigations are warranted to clarify different aspects of the association between these risk factors, especially new ones (e.g., psychological stress, intestinal microbiota), and the risk of CRC.

CRC prevention methods may be classified as “primary” and “secondary” prevention. Primary prevention includes methods for reduction in or elimination of exposure to risk factors (e.g., strategies for reducing smoking and alcohol consumption) as well as strategies for increasing protective factors (e.g., physical activity, dietary fiber, aspirin). The secondary prevention method, CRC screening, includes diagnosis and removal of colorectal adenomatous polyps. A number of methods have been proposed for CRC screening, including the FOBT (guaiac test/FIT test), the stool DNA test, lower-GI endoscopy (sigmoidoscopy/colonoscopy), and CTC. Considering a combination of screening methods (e.g., FOBT + colonoscopy) and targeting eligible individuals according to the levels of exposure to risk factors (risk-based strategy) will increase the accuracy and effectiveness of CRC screening programs. The development of CRC screening guidelines is a complex process, and different factors should be mentioned in this process, including the accuracy of screening methods (e.g., sensitivity and specificity) and the characteristics of target populations (e.g., patient preferences, availability of screening modalities, costs, benefits, and adverse events). According to the ACS guideline, average-risk individuals are suggested to start CRC screening at 45. The ACS recommended a combination of screening methods at specific intervals, including FOBT, stool DNA test, sigmoidoscopy, CTC, and colonoscopy. According to the ACP guideline, CRC screening should start at age 50. The ACP also suggest multiple screening methods at specific intervals, including the FOBT, sigmoidoscopy, and colonoscopy.

Considering effective risk reduction strategies as well as the design and successful implementation of population-specific CRC screening programs has a pivotal role in CRC control programs in each community. High-quality population-specific research is needed to select the best and most accurate and effective methods for the primary prevention and screening of CRC in target populations.
